# Spatio-Temporal Trends and Identification of Correlated Variables with Water Quality for Drinking-Water Reservoirs

**DOI:** 10.3390/ijerph121013179

**Published:** 2015-10-20

**Authors:** Qing Gu, Ke Wang, Jiadan Li, Ligang Ma, Jinsong Deng, Kefeng Zheng, Xiaobin Zhang, Li Sheng

**Affiliations:** 1 Institute of Digital Agriculture, Zhejiang Academy of Agricultural Sciences, Hangzhou 310021, China; E-Mails: funny@zju.edu.cn (Q.G.); riceipm1@zju.edu.cn (X.Z.); LLANDLL070701@163.com (L.S.); 2 Institution of Remote Sensing and Information System Application, Zhejiang University, Hangzhou 310058, China; E-Mails: kwang@zju.edu.cn (K.W.); jsong_deng@zju.edu.cn (J.D.); 3 Institute of Rural Development and Information, Ningbo Academy of Agricultural Sciences, Ningbo 315040, China; E-Mail: vaneljd@163.com; 4 College of Resource and Environmental Science, Xinjiang University, Urumqi 830046, China; E-Mail: mosanzju@126.com; 5 School of Civil Engineering and Environmental Sciences and School of Meteorology, University of Oklahoma, Norman, OK 73019, USA

**Keywords:** water quality, reservoir, spatial distribution, temporal variation, cluster analysis

## Abstract

It is widely accepted that characterizing the spatio-temporal trends of water quality parameters and identifying correlated variables with water quality are indispensable for the management and protection of water resources. In this study, cluster analysis was used to classify 56 typical drinking water reservoirs in Zhejiang Province into three groups representing different water quality levels, using data of four water quality parameters for the period 2006–2010. Then, the spatio-temporal trends in water quality were analyzed, assisted by geographic information systems (GIS) technology and statistical analysis. The results indicated that the water quality showed a trend of degradation from southwest to northeast, and the overall water quality level was exacerbated during the study period. Correlation analysis was used to evaluate the relationships between water quality parameters and ten independent variables grouped into four categories (land use, socio-economic factors, geographical features, and reservoir attributes). According to the correlation coefficients, land use and socio-economic indicators were identified as the most significant factors related to reservoir water quality. The results offer insights into the spatio-temporal variations of water quality parameters and factors impacting the water quality of drinking water reservoirs in Zhejiang Province, and they could assist managers in making effective strategies to better protect water resources.

## 1. Introduction

The water scarcity in China has become a serious issue affecting socio-economic development, not only because of the insufficient and disproportionate distribution of water resources as well as a dense population, but also because of the inefficient regulation and management of water resources [1,2]. Naturally, the spatio-temporal distribution of water resources is inconsistent with the increasing socio-economic needs for water, resulting in conflicts between water supply and demand. Dramatic urbanization and industrialization leads to increasingly severe deterioration of surface and ground water [2,3]. Unfortunately, poor water resource management has made the situation worse, increasing China’s vulnerability to water shortages [4,5,6]. Moreover, water crises in the future may not be caused by physical scarcity of water but would more likely be caused by inadequate or inappropriate water governance [7].

One critical step to effectively managing water resources is the development of a water monitoring network, which is a vital element in water restoration and protection efforts [8]. Long-term water quality monitoring programs result in a large data matrix comprising physical properties and nutrient, inorganic, and biological parameters [3,9]. However, due to the latent interrelationships between parameters or monitoring sites, as well as the abundances and complexities of the monitoring data, it is difficult to analyze, interpret, and extract valuable information from these data [9,10]. Thus, it is an important requirement to extract meaningful information from these data for further policy making using a variety of approaches to characterize spatio-temporal trends, to discriminate significant parameters, and to identify the correlated variables with water quality variability [10,11,12]. 

The evaluation of water quality in most countries has become a critical issue in recent years, especially in those countries, such as China, whose fresh water resources are deficient [2,13]. Previous studies often focused on the water in rivers or lakes [3,14,15,16], and few studies have examined the spatial and temporal variability of reservoir water quality. Along with rapid economic development and urban expansion, reservoir water quality was also confronted with increasing pollution pressure and degradation, as indicated by the monitoring data. Thus, it is urgent that meaningful information about the status of the drinking water reservoirs be obtained using practicable methods.

Drinking-water reservoirs play a critical role in the sustainable development of Zhejiang Province, which is a fairly economically-developed and highly-urbanized area in China. Several reports have been published on the assessment of chlorophyll-a concentrations, heavy metal concentrations, and eutrophication conditions of single or small numbers of reservoirs [17,18,19], but no water quality evaluation has been carried at the provincial scale using advanced mathematical methods (e.g., statistical analysis and pattern recognition). Owing to sufficient data and effective approaches, this paper evaluated the water quality of 56 drinking water reservoirs and attempted to explore the spatio-temporal trends of water quality as well as the correlated variables with water quality variability in reservoirs.

More specifically, this paper focuses on the following main objectives: to evaluate and analyze the spatial and temporal trends in water quality for 56 drinking water reservoirs in Zhejiang Province; to identify the parameters that are most closely related to water quality; and to analyze the relationships between the correlated variables and reservoir water quality.

## 2. Study Area and Materials

### 2.1. Study Area

Zhejiang Province is located in the eastern coastal area of China, and covers approximately 101,800 km^2^ and had a population of 54.77 million in 2012. As one of the most economically-developed regions in China, Zhejiang is also a densely populated province. The terrain in Zhejiang is characterized by complex topography consisting of 70.4% mountains and hills, 23.2% plains and basins, and 6.4% rivers and lakes. The climate is subtropical monsoon, with an annual average temperature of 17.8 °C and an annual average rainfall between 980 and 2000 mm.

**Figure 1:**
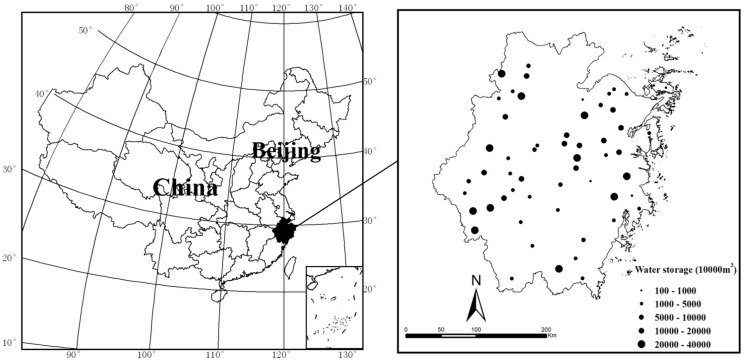
Location of the study area, and the spatial distribution of study reservoirs.

Surface water reservoirs have functioned for many years as the most important drinking water resource in Zhejiang Province. Approximately 500 drinking-water reservoirs have been supporting 70% of the resident population in Zhejiang. The reservoirs have been gradually taking responsibility for fresh water supply, mainly due to the increasing deterioration of river water quality. Considering the spatial distribution of reservoirs, as well as data availability, we selected 56 representative and typical reservoirs for study. Figure 1 shows the location of the study area and the spatial distribution and sizes of the reservoirs investigated.

### 2.2. Data Collection

Four water quality parameters (total nitrogen (TN), total phosphorus (TP), five day biochemical oxygen demand (BOD), and chemical oxygen demand (COD)) from 56 drinking water reservoirs over five years (2006–2010) were obtained for the study. All of the records were gathered from local monitoring stations by the Zhejiang Environmental Protection Bureau. We chose only the above four parameters for study because they were widely used in previous investigations and were the most important pollution parameters for reservoirs in Zhejiang.

The other data used in this study includes: a digital land use map for the year 2010 provided by the Chinese Ministry of Environmental Protection. The dataset was retrieved from the interpretation of remote-sensing data and field surveys with a validated overall accuracy exceeding 90%; a digital elevation model (DEM) with 30 m resolution, administrative divisions, a drainage map, and socio-economic statistics provided by the Environmental Science Research Institute in Zhejiang Province. The land use types were classified into five categories: forests, arable land, impervious areas, water bodies, and unused land. Arable land included paddies and upland agricultural fields. Impervious areas covered all areas of impervious surfaces, including residential areas, commercial areas, and roads. The DEM, assisted by the drainage map, was used to delineate the boundaries of watersheds using the Hydrology module in ArcGIS 9.3. The land use shape file was then overlaid by the watershed boundary layer to compute land use parameters within the watersheds.

## 3. Methodology

### 3.1. Cluster Analysis (CA)

CA is an unsupervised pattern recognition technique covering a wide variety of algorithms for delineating natural groups or clusters based on their nearness or similarity [20,21]. Hierarchical clustering is the most widely used algorithm. By starting with the most similar object pairs and generating clusters in step-by-step structures, a data set can be sequentially classified into categories or clusters [13]. The results of hierarchical clustering are usually presented in a dendrogram [22,23]. In our analysis, hierarchical agglomerative CA (HACA) was performed, using Ward’s method with squared Euclidean distances as a measure of similarity between two samples. HACA was employed for the reservoir water quality data set to group the spatially similar sampling reservoirs for further spatial distribution analysis. The HACA process was performed using IBM SPSS Statistics 20 (SPSS/IBM, Chicago, IL, USA).

### 3.2. Variables Potentially Correlated with Water Quality

Based on the comprehensive analysis of the characteristics of the study area and on the availability, comparability, and reliability of the data, we selected 10 variables, including four categories (land use, socio-economic factors, geographic features, and reservoir attributes) to perform correlation analysis with water quality parameters.

It is well-known that anthropogenic activities have distinct influence on adjacent water bodies because of the discharge of various pollutants, such as domestic sewage and industrial waste [3,24]. In this study, we selected resident population, gross domestic product (GDP), and gross industrial output value as variables. The resident population was referenced to the Sixth China Population Census. The GDP and gross industrial output value were extracted from the Zhejiang Statistical Yearbook 2010 [25] and were collected at the county scale. Considering the close connection between these parameters and the impervious areas, we calculated the corresponding amount of each of the above variables for each watershed according to the proportion of impervious areas in the watershed to that in the administrative region.

Land use has been demonstrated to significantly impact the water quality of adjacent aquatic systems by influencing the hydrologic processes in the watersheds [26,27]. Many water quality parameters in various water systems showed close relationships with the land use indicators within watersheds [27,28]. In this paper, we selected forests (%), arable land (%), and impervious areas (%) to represent the land use characteristics in reservoir watersheds.

Elevation and distance to city, as well as storage capacity and age, were selected to explore their relationships with reservoir water quality parameters. Geographical position influences the way that pollutants get into water systems, probably resulting in water quality variations [29,30]. The elevations of the reservoir locations were employed to represent the geographical positions of the reservoirs. Previous studies have discussed the impact of cities on aquatic environments, finding that cities have negative effects on water quality [31,32]. We calculated the distances between each study reservoir and the nearest city center to analyze the influence of cities on water bodies. Different storage capacities or ages could lead to discrepancies in purification ability, environmental capacity, and risk of contamination [33,34].

## 4. Results and Discussion

### 4.1. Descriptive Statistics of Studied Parameters

Summary statistics for the selected variables and water quality parameters are presented in Table 1. The mean surface area, the mean watershed area, and the mean storage capacity of the 56 studied reservoirs were 2.35 km^2^, 129.74 km^2^, and 72.25 million m^3^, respectively. The mean age of the reservoirs was 31.27 years, and all of the study reservoirs were constructed during the period 1950–2010. The mean proportion of impervious areas in the watersheds was only 3.29%, and the mean proportions of arable land and forest were 9.5% and 77.96%, respectively. The standard deviations of impervious areas and arable land percentages were 3.49 and 7.69, respectively, and the proportion of forest land varied substantially from 7.29% to 98.98% with a S.D. of 16.97. Substantial differences between the minimum and maximum values were observed for all of the socio-economic parameters, indicating appreciable regional variances. Each water quality parameter exhibited a wide variation, as indicated by large standard deviations in the data set. In reference to the environmental guidelines of the national quality standards for surface waters, China (GB 3838-2002) (Appendix Table A1), the mean value of TN reached the fourth level of the surface water quality standard, the mean value of TP reached the third level, and those of BOD and COD reached the first level of the standard. The maximum values of TN and TP exceeded the fifth level of the standard, the maximum BOD exceeded the fourth level, and the maximum COD reached the third level of the standard. 

**Table 1:** Descriptive statistics of the studied parameters.

Categories	Measures	Min.	Max.	Mean	S.D.
Reservoirs	Surface area (km^2^)	0.11	12.35	2.35	2.51
	Watershed area (km^2^)	1.82	728.39	129.74	148.68
	Storage capacity (10,000 m^3^)	123	30250	7224.63	7863.27
	Age (years)	3	63	31.27	16.68
Land use	Impervious areas (%)	0	27.02	3.29	3.49
	Arable land (%)	0.08	45.01	9.5	7.69
	Forests (%)	7.92	98.98	77.96	16.97
Socio-economic factors	Resident population (10,000 people)	8.13	123.2	51.13	26.31
	GDP (0.1 billion yuan)	23.1	1044.85	240.57	179.98
	Gross industrial output value (0.1 billion yuan)	2.23	454.80	84.02	84.39
Geographical features	Elevation (m)	12	464	120.51	100.35
	Distance to city (km)	0.3	22.8	15.79	12.65
Water quality	BOD (mg/L)	0.62	7.95	2.57	1.17
	COD (mg/L)	1.92	18.77	6.85	3.73
	TN (mg/L)	0.11	5.69	1.12	0.69
	TP (mg/L)	0.01	0.33	0.05	0.06

### 4.2. Spatial Distribution

CA was applied to the selected four water quality variables to detect similarity among the sampling sites (spatial variability). After data normalization by z-transformation, HACA was performed using Ward’s method of linkage with squared Euclidean distance as a measure of similarity [13]. The significance of the groups obtained was tested by Sneath’s index of disjunction [35]. 

**Figure 2:**
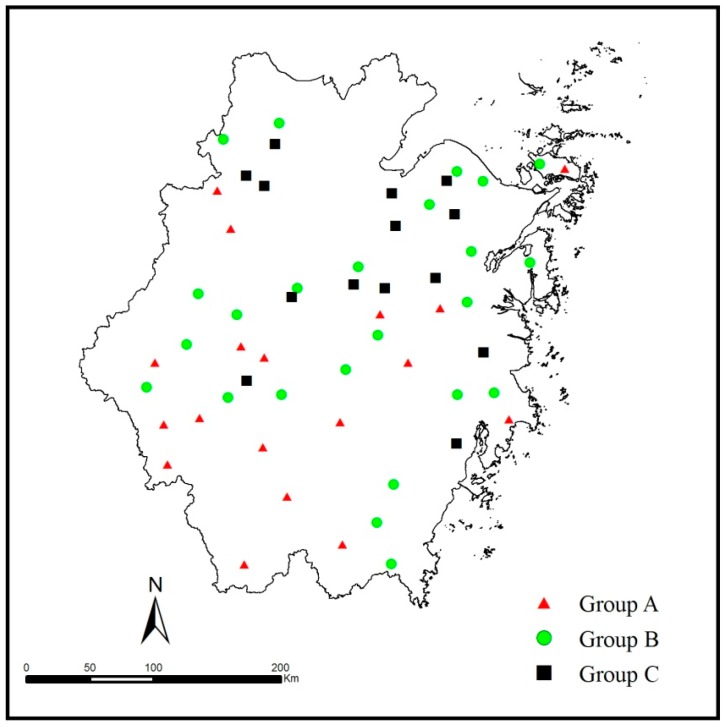
The spatial distribution of reservoir water quality.

The 56 reservoirs were grouped into three statistically significant clusters (A, B and C), as shown in Figure 2. Groups A, B, and C consisted of 18, 24, and 14 reservoirs, respectively. The group classifications varied significantly because the sites within each group had similar natural backgrounds that were influenced by similar factors. As shown in the diagram, the reservoirs in Group A, which had the best water quality, were primarily located in the southwest region, corresponding to low-pollution regions. The reservoirs in Group B, with moderate water quality, were dispersed throughout the provincial area and were located in moderate-pollution regions. The reservoirs in Group C, corresponding to reservoirs that had relatively poor water quality conditions, were distributed in regions of high pollution, where they most likely received pollution from urbanization and industrialization. By inspecting the spatial distribution of samples, water quality exhibited a trend of degradation from southwest to northeast.

**Figure 3:**
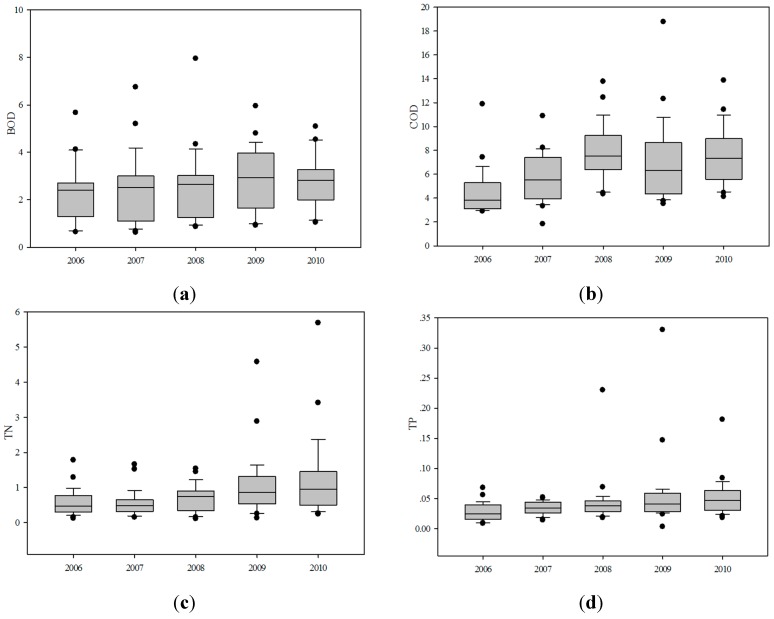
Temporal variations of (**a**) BOD; (**b**) COD; (**c**) TN and (**d**) TP in the study reservoirs from 2006 to 2010 (units: mg/L).

### 4.3. Temporal Trends

Box and whisker plots were used to represent the temporal trends of the four parameters. As we can see from Figure 3, the average concentration of BOD was relatively stable during the first three years, and was highest in 2009, followed by 2010. The average concentration of COD had a significant increase from 2006 to 2008, declined in 2009, and rose again in 2010. The average concentration of TN was lowest in 2007, and this was followed by a notable rise, with the concentration in 2010 being significantly higher than in 2007. The trend of TP was somewhat similar to TN, which showed a steady increase during the five years, indicating increasingly serious eutrophication. TN and TP both showed some dispersed points that denoted very high concentration.

### 4.4. Identification of Correlated Variables with Water Quality

#### 4.4.1. Relationship between the Selected Variables and Water Quality Parameters

The relationships between the selected variables and reservoir water quality parameters were analyzed using correlation analysis. In line with the land use data, we chose the water quality records in 2010 and collected the socio-economic data of 2010 for computation. The results of Pearson correlation analysis are listed in Table 2. Land cover indicators and socio-economic indicators showed significant correlation with water quality parameters, especially with regard to proportion of forest, proportion of impervious areas, GDP, and gross industrial output value. However, regarding geographical features and reservoir attributes, only distance to city was negatively correlated with BOD. All of the above variables, except for proportion of forest and distance to city, showed positive relationships with water quality parameters. In particular, the relationships between water quality parameters and land use indicators (as well as socio-economic data) are discussed in the following sections.

**Table 2:** Correlation analysis between independent variables and water quality parameters.

	Arable Land (%)	Forests (%)	Impervious Areas (%)	Resident Population (10,000 People)	GDP (0.1 Billion Yuan)	Gross Industrial Output Value (0.1 Billion Yuan)	Elevation (m)	Distance to City (km)	Storage Capacity (10,000 m^3^)	Age (Years)
TN	0.08	−0.25 **	0.14	0.28 **	0.26 **	0.52 **	0.11	−0.09	−0.05	0.03
TP	0.12	−0.07	0.21 *	0.19 *	0.13	0.19 *	−0.02	−0.05	0.11	−0.04
BOD	0.04	−0.20 *	0.58 **	0.12	0.21 *	0.59 **	−0.02	−0.21 *	0.07	−0.07
COD	0.19 *	−0.31 **	0.51 **	0.31 **	0.09	0.29 **	0.07	0.04	−0.06	0.10

Notes: * *p* < 0.05, ** *p* < 0.01; *n* = 56.

#### 4.4.2. Land Use Types and Water Quality

Many studies have demonstrated a strong relationship between land use and the water quality in adjacent water bodies [26,28,36,37]. Correlation analysis in this study revealed interesting aspects of the effects of land use types within the watersheds on reservoir water quality. As we can see from Table 2, the water quality parameter COD had a significant positive correlation with the proportion of arable land in watersheds, but the other three water quality parameters showed no significant correlations with the proportion of arable land. Although these results are inconsistent with a number of previous studies that found arable land use to be an important contributor to water quality deterioration [37,38], they are similar to the result of [28]. BOD and COD showed very significant positive relationships with the proportion of impervious areas in watersheds, and TP was also correlated with it. This is in agreement with the findings in previous studies where the expansion of impervious areas contributed to increased risks of degraded water quality [36,38]. The proportion of forests was strongly negatively correlated with BOD, COD, and TN, but showed no significant relationship with TP. This can be ascribed to the positive contribution of forest to water conservation and purification [37].

Our results were consistent with previous studies indicating better water quality levels in reservoirs with smaller proportions of impervious areas and higher proportions of forest in their watersheds [36,37,38]. However, a relatively weak relationship was found between the proportion of arable land and water quality parameters. It can be inferred that the negative influence that agricultural activities have on water bodies is closely related to tillage practices and geographic features [39,40]. In Zhejiang Province, paddy fields were the main type of arable land, and the farming methods of paddies are different from those for other crops in many respects, such as fertilizing frequency and field water management [37,38]. To facilitate fertilizer absorption by rice plants, farmers will keep the fields flooded after fertilizing. Therefore, the loading of nutrients such as nitrogen and phosphorus from paddies to reservoirs is largely determined by water management and rainfall [40,41]. Large amounts of rainfall will lead to overflow from paddy fields, carrying nutrients and adversely affecting reservoir water quality [40]. In addition, agricultural non-point source pollution usually takes place on sloping land, significantly contributing to water quality degradation [42]. Slope is another important factor that relates to the degree of negative impact of arable land on water quality in adjacent water bodies [43]. The risk of area-source pollution is modest in plain regions, but is intensified in sloping cropland. The differences in tillage management and annual rainfall, as well as the local slope, may jointly account for the impacts of arable land use on reservoir water quality. Thus, the proportion of arable land area within watersheds does not linearly correlate with the reservoir water quality parameters.

#### 4.4.3. Socio-Economic Variables and Water Quality

It has been demonstrated that the water quality of drinking water reservoirs is mainly related to socio-economic situations [44]. These socio-economic sources of pollution mainly include domestic sewage, solid wastes, and industrial wastewater discharge, which can be indicated by socio-economic data such as GDP, gross industrial output value and population [45]. As shown in Table 2, TN, TP, and COD had significant positive relationships with resident population, but BOD showed no significant correlation with it. TN and BOD had positive relationships with GDP. The gross industrial output value was very significantly positively correlated with TN, BOD, and COD, and was significantly correlated with TP.

**Figure 4:**
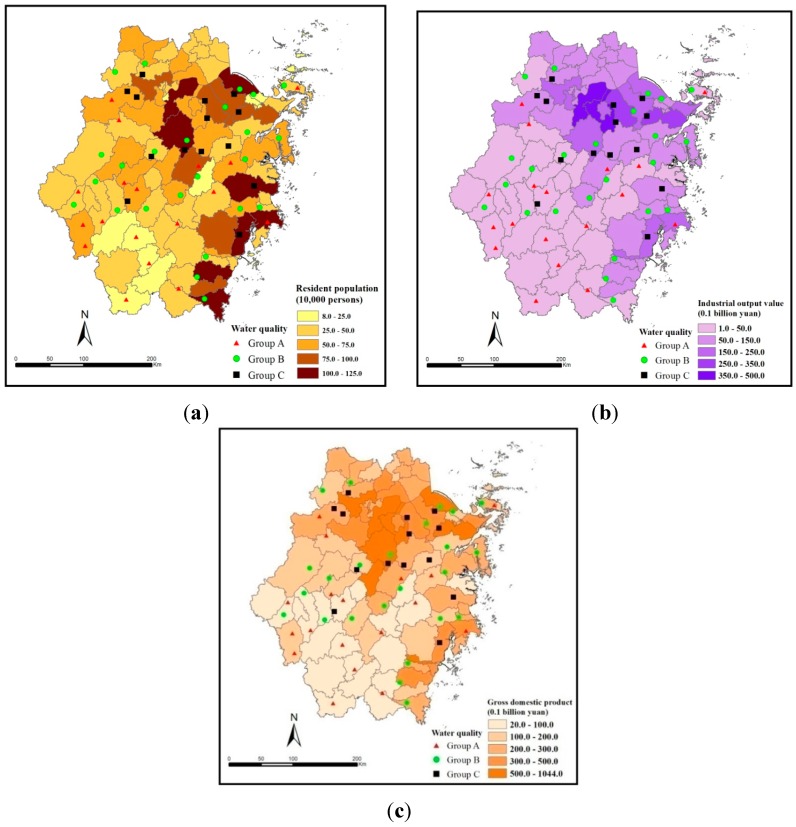
Spatial distributions of the selected socio-economic indicators at the county level of Zhejiang Province in 2010: (**a**) resident population; (**b**) gross industrial output value; (**c**) GDP.

**Figure 5:**
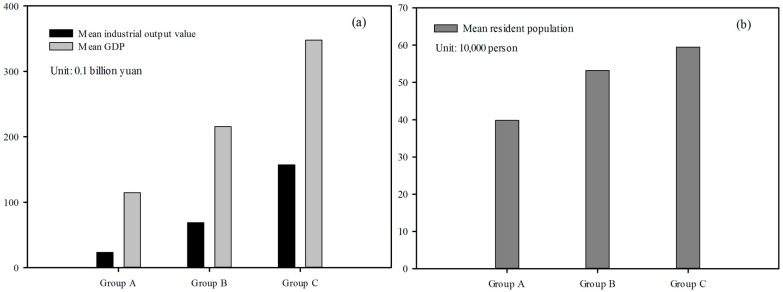
Mean values of the selected socio-economic indicators in Group A, B, and C: (**a**) mean gross industrial output value and mean GDP; (**b**) mean resident population.

The indicators at the county level of Zhejiang Province in 2010 were chosen to verify the spatial consistency between reservoir water quality and regional socio-economic status, as shown in Figure 4. Evidently, the reservoir water quality conditions were spatially consistent with the socio-economic indicators. The overall reservoir water quality degraded from the southwest to the northeast, which corresponded to the socio-economic situation of the region. As we can see from Figure 5, socio-economic status clearly increases pressure on adjacent waters, because the water quality levels of reservoirs located in areas with higher populations, GDP and gross industrial output value were inferior to those of reservoirs in less developed areas.

**Figure 6:**
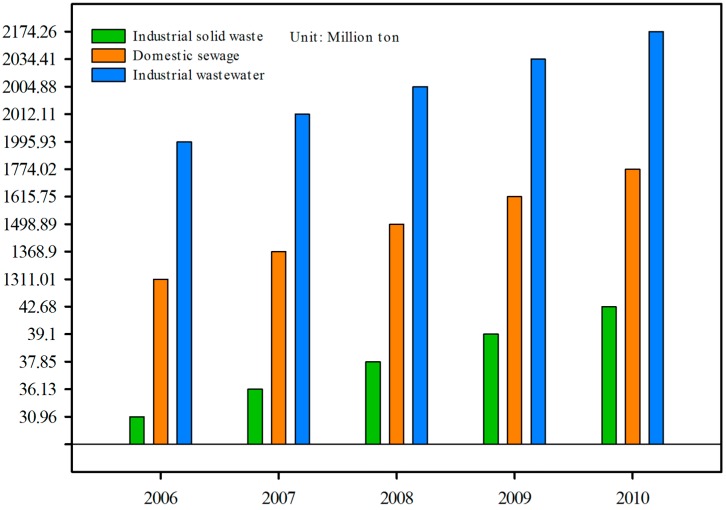
The industrial wastewater, industrial solid waste, and domestic sewage emissions in Zhejiang Province between 2006 and 2010.

Zhejiang Province has experienced drastic urbanization and industrialization in the past decades, which has been accompanied by various environmental problems due to the absence of environmental awareness. According to the statistical records [25], the industrial wastewater, industrial solid waste, and domestic sewage emissions substantially increased from 2006 to 2010 (Figure 6), especially in the relatively developed cities in the northern and eastern areas of the province, such as Hangzhou, Ningbo, Shaoxing and Wenzhou. However, due to the high cost of treatment of pollutants and the lack of strict enforcement of laws, a substantial amount of pollutants was directly discharged into adjacent environments and was probably carried by surface runoff into reservoirs. This dire tendency, if not controlled, would give rise to devastating consequences of environmental deterioration and a water insecurity crisis. From a policy perspective, much more aggressive enforcement of environmental law is necessary and urgent, as is real-time monitoring of reservoir water quality and environmental conditions in watersheds. Especially in those rapidly developing regions, maintaining natural water resources during urbanization has become one of the most pressing missions. It is necessary to strictly control the populations within watersheds to alleviate the pressure from domestic pollutants on reservoirs. Industrial development must be aggressively controlled and supervised, and factories posing pollution risks must be prohibited within reservoir watersheds [46].

## 5. Conclusions

Reservoirs have been serving for many years as the most important sources of drinking water in some regions in China. This study contributes to the assessment of spatio-temporal trends in water quality parameters as well as factors influencing water quality in drinking water reservoirs. The proposed data analysis framework in this study is feasible and effective, and is applicable to other areas, serving as an operational analysis tool for managers to better formulate strategies against water degradation.

The results of this study indicated that the reservoir water quality spatially degraded from the southwest to the northeast, which corresponded to the socio-economic situation of the region. The temporal evaluation showed that the overall water quality condition deteriorated from 2006 to 2010. According to the correlation analysis, land use and socio-economic indicators were most closely correlated with the reservoir water quality parameters. The water quality parameter COD was positively correlated with the proportion of arable land in watersheds, and BOD, COD, and TP showed significant positive relationships with the proportion of impervious areas in watersheds. BOD, COD, and TN were strongly negatively correlated with the proportion of forest land use. TN, TP, and COD had significant positive relationships with resident population, and TN and BOD had positive relationships with GDP. The gross industrial output value was strongly positively correlated with all four water quality parameters. These results may provide a useful resource for improving strategy formulation and reservoir water quality protection by planners and managers.

## Appendix

**Table A1:** Environmental guideline of national quality standards for surface waters, China (GB3838-2002) (units: mg/L).

Parameters		Category of Water Quality Standards				
		First	Second	Third	Fourth	Fifth
DO	≥	7.5	6	5	3	2
COD_Mn_	≤	2	4	6	10	15
COD	≤	15	15	20	30	40
BOD	≤	3	3	4	6	10
NH3-N	≤	0.15	0.5	1	1.5	2
TP	≤	0.01	0.025	0.05	0.1	0.2
TN	≤	0.2	0.5	1	1.5	2
TCu	≤	0.01	1	1	1	1
TZn	≤	0.05	1	1	2	2
F^−^	≤	1	1	1	1.5	1.5
TSe	≤	0.01	0.01	0.01	0.02	0.02
TAs	≤	0.05	0.05	0.05	0.1	0.1
THg	≤	0.00005	0.00005	0.0001	0.001	0.001
TCd	≤	0.001	0.005	0.005	0.005	0.01
Cr^6+^	≤	0.01	0.05	0.05	0.05	0.1
TPb	≤	0.01	0.01	0.05	0.05	0.1
TCN	≤	0.005	0.05	0.2	0.2	0.2
V-ArOH	≤	0.002	0.002	0.005	0.01	0.1
Petroleum	≤	0.05	0.05	0.05	0.5	1
Anionic surfactant	≤	0.2	0.2	0.2	0.3	0.3
S^2−^	≤	0.05	0.1	0.05	0.5	1
Fecal coliform (number/L)	≤	200	2000	10,000	20,000	40,000
